# Motor Imagery Under Distraction— An Open Access BCI Dataset

**DOI:** 10.3389/fnins.2020.566147

**Published:** 2020-10-19

**Authors:** Stephanie Brandl, Benjamin Blankertz

**Affiliations:** ^1^Department of Machine Learning, Technische Universität Berlin, Berlin, Germany; ^2^Department of Neurotechnology, Technische Universität Berlin, Berlin, Germany

**Keywords:** brain-computer interface, motor imagery, out-of-lab scenarios, artifacts, steady-state visual evoked potential (SSVEP), vibro-tactile stimulation

## 1. Introduction

Research studies in the field of Brain-Computer Interfaces (BCI) mostly take place in controlled lab environments. To move BCIs into the real world and everyday life situations it is crucial to bring research out of those controlled environments and into more realistic scenarios.

Recently, various studies have been recorded in classrooms, cars or realistic tugboat simulators (Blankertz et al., 2010; Brouwer et al., 2017; Ko et al., 2017; Miklody et al., 2017). Mobile BCIs even allow participants to move freely during the recording (Lotte et al., 2009; Castermans et al., 2011; De Vos et al., 2014; Wriessnegger et al., 2017; von Lühmann et al., 2019). Other studies have been carried out with paralyzed, locked-in or completely locked-in users or with participants recovering from stroke (Neuper et al., 2003; Ang et al., 2011; Leeb et al., 2013; Höhne et al., 2014; Hwang et al., 2017; Han et al., 2019; Lugo et al., 2020).

However, so far there has not been a BCI study where distractions are investigated systematically. We have recorded a motor imagery-based BCI study (*N* = 16) under five types of distractions that mimic out-of-lab environments and a control task where no distraction was added. The secondary tasks include watching a flickering video, searching the room for a specific number, listening to news, closing the eyes and vibro-tactile stimulation.

Many BCI datasets have been published, e.g., in context of the BNCI Horizon 2020 initiative^1^, 4 BCI competitions have had a big impact on the research community (Sajda et al., 2003; Blankertz et al., 2004, 2006; Tangermann et al., 2012) and still datasets are made available (Shin et al., 2016; Cho et al., 2017; Kaya et al., 2018). We want to contribute further by publishing this BCI dataset with multiple distractor conditions. This report provides a summary of the design and experimental setup of the study. We also show group-level results on event-related synchronization and desynchronization, results on a standard classification pipeline and power spectra for all secondary tasks. Apart from the dataset^2^, code for the analysis is also publicly available^3^ and a more advanced analysis can be found in Brandl et al. (2016).

## 2. Methods

### 2.1. Participants

Sixteen participants (six female, average age 26.3 ± 1.9 years) volunteered to participate in this study. Three volunteers had previously participated in another BCI experiment. All instructions were given in German requiring basic language skills. Volunteers were reimbursed for their participation in the study except for three employees of the TU Berlin Machine Learning Group. All participants were instructed on the experimental procedures prior to signing an informed consent. This study was conducted according to the declaration of Helsinki and was approved by the Ethics Committee of the Charite-Universitätsmedizin Berlin (approval number: EA4/012/12).

### 2.2. Data Acquisition

EEG signals were recorded with a Fast'n Easy Cap (EasyCap GmbH) with 63 wet Ag/AgCl electrodes which were placed at symmetrical positions according to the international 10–20 system (Jasper, 1958) referenced to the nose. We used two 32-channel amplifiers (BrainAmp, BrainProducts) to amplify the signals, which were sampled at 1,000 Hz. Data was recorded in the period of 15 April–18 July 2014 at TU Berlin and raw data without any preprocessing was made publicly available^1^.

### 2.3. Experimental Setup

During the experiment, the participants were sitting in a comfortable armchair at a distance of 1m in front of a 24” computer screen. Auditory instructions were given via headphones.

Each experimental session lasted about 3 h including preparation and about 90 min of signal recording. Before the main experiment, we recorded eight trials in which participants had to alternately keep their eyes open or closed for 15 s.

The main experiment was divided into seven runs à 10 min with 72 trials per run. One trial lasted 4.5 s and was defined by one motor imagery task with an additional secondary task except for the first run. The first run served as a calibration phase without feedback and distraction tasks. The subsequent runs included three blocks à four trials (two left and two right) of each secondary tasks (72 trials per run). The blocks were presented in a random order to minimize sequence effects.

#### 2.3.1. Primary Task

At the beginning of each trial, instructions for left or right hand motor imagination were given over headphones (*links* and *rechts* as the instructions were in German). This was the primary task in this study. At the end of the trial the participant received a *stop* command followed by a break of 2.5 s, after which the next trial started.

Participants were asked to choose one haptic hand movement. Several strategies for motor imagery were presented to the participants to choose from. The majority chose to imagine squeezing a soft ball—other strategies involved opening a water tap, piano playing or using a salt shaker.

Auditory online feedback was given in the six runs after the calibration to keep the motivation up. The online feedback was trained on the calibration data and based on Laplacian filters of the C3 and C4 electrodes (McFarland et al., 1997) and regularized linear discriminant analysis (RLDA, Friedman, 1989). For this, EEG data was downsampled to 100 Hz, Laplacian filters of C3 and C4 were calculated and the data was band-pass filtered in the ranges 9–13 and 18–26 Hz with a Butterworth filter of order 5. Data was then cut into epochs of 750–3,500 ms and an RLDA classifier was trained on the logarithm of variances as features. During the feedback phase, EEG data was downsampled and band-pass filtered as before, projected on the Laplacian filters and the trained classifier applied on the log-variance features. Furthermore, we applied pooled-mean adaptation to continue training the classifier during the feedback phase (Vidaurre et al., 2010). Classification averaged across all participants reached an accuracy of 57.05%. Auditory feedback was given after the *stop* command as *decision left* (*Entscheidung links*) or *decision right* (*Entscheidung rechts*) during the 2.5 s break. Online classification was performed with the BBCI toolbox in MATLAB^4^.

#### 2.3.2. Secondary Tasks

We simulated a pseudo-realistic environment by adding six secondary tasks on top of the primary motor imagery task to the experimental setup. They were selected to cover different types of distractions in an out-of-lab scenario.

1. Clean
  This condition served as a control task where no additional distraction was added.
2. Eyes-Closed
  Participants were asked to close their eyes before the motor imagery trial started and to keep them closed until the trial finished. Here, we expected a power increase in the alpha band (8–12 Hz) due to the closed eyes to overlap with the motor task related mu rhythm (8–13 Hz). This task was also the primary reason for providing all instructions and feedback auditorily instead of visually.
3. News
  Short sequences of a public newscast (*Tagesschau*) were played over the headphones with current news (January/February 2014) and news from 1994. Each sequence was only played once in each experiment. We expected the participants to be cognitively distracted and the auditory cortex to be activated during the motor imagery task which might influence the motor imagery performance.During the experiment, we did not assess active listening of the participants.
4. Numbers
  For this task, 26 sheets of paper with a randomly mixed letter-number combination were set up on the wall in front of the participants and also on the left and right side of the room. This implies that participants needed to turn their head in order to see the sheets. For each trial a new window appeared on the screen asking the participants to search the room for a particular letter to match with a stated number and to read it out loud. Each combination was shown 2–3 times to all participants. We counted how often the letters were found. Out of 72 trials, 59.7 combinations were successfully found on average. This task was expected to cause both a high cognitive distraction and additional muscular artifacts.
5. Flicker
  A flickering stimulus with alternating gray shades at a frequency of 10 Hz was presented on the screen. We included this task to analyze the influence of the *steady state visually evoked potential* (SSVEP) (Morgan et al., 1996).
6. Stimulation
  We placed two coin vibration motors with a diameter of 3 cm on the insides of both forearms, one over each wrist and the other just below the elbows. To investigate the interference of *steady state vibration somatosensory evoked potential* (SSVSEP, Tobimatsu et al., 1999; Brouwer and Van Erp, 2010) on the motor imagery task, vibrotactile stimulation was carried out with carrier frequencies of 50 and 100 Hz, each modulated at 9, 10, and 11 Hz.

### 2.4. Baseline Analysis

**Figure 1:**
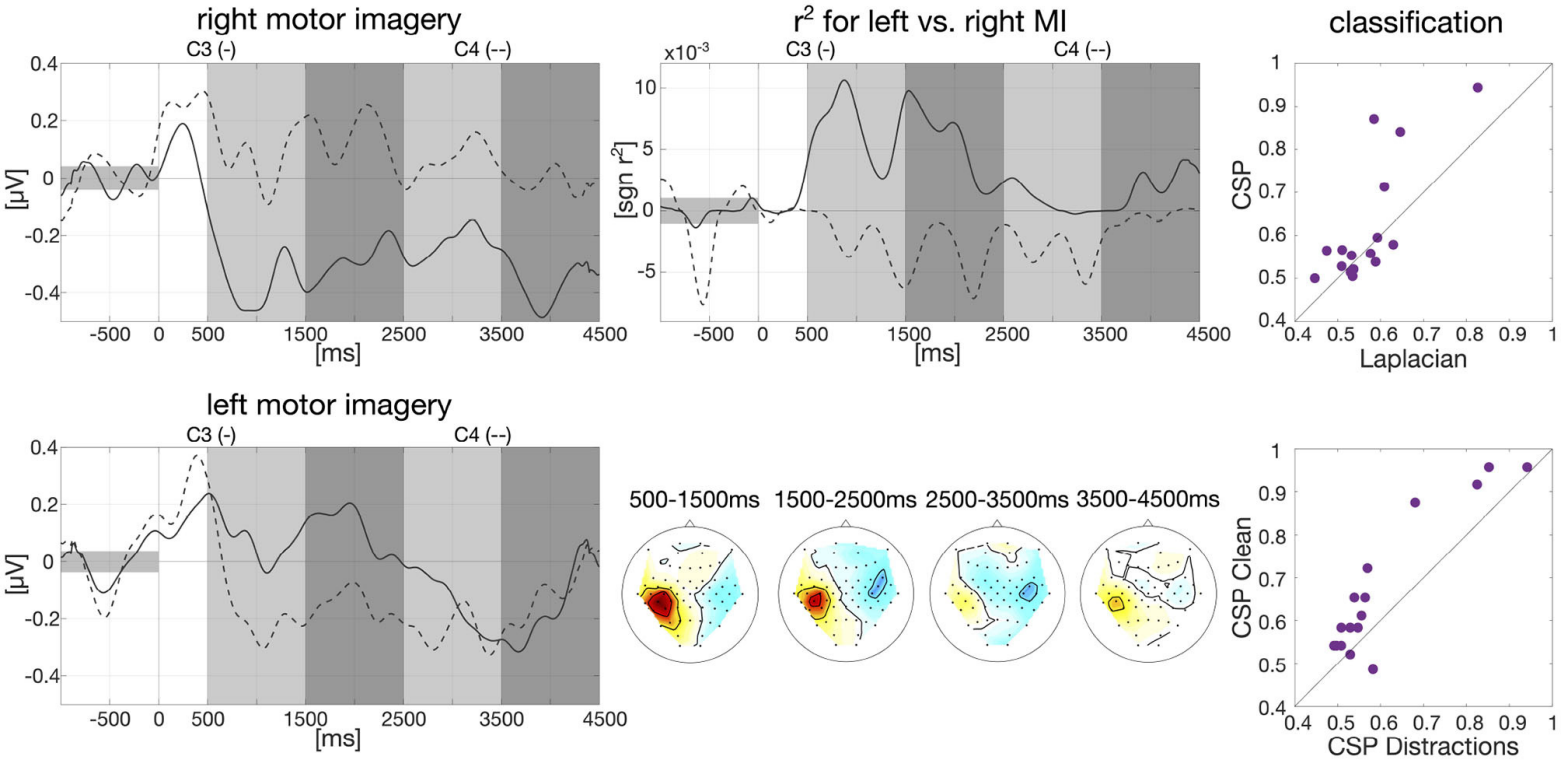
Baseline analysis for calibration data and first classification results. Left: Group-average envelopes of C3 (solid) and C4 (dashed) electrodes for right and left motor imagery trials. Center: Group-average signed *r*^2^ values evolving over time within trials as times series for C3 and C4 (upper) and patterns for four different intervals (lower). Right: Classification results on motor imagery data left vs. right (one dot per participant): comparison offline CSP vs. online Laplacian (upper) and comparison CSP without distractions vs. CSP with distractions (lower).

We show group-level results of event-related synchronization and desynchronization (ERS/ERD, see Figure 1) which can be observed during motor imagination and execution (Pfurtscheller, 1992). Data analysis was also performed with the BBCI toolbox for MATLAB^4^.

Data from the calibration session was band-pass filtered in the frequency band of 9–13 Hz with a 3rd order zero-phase Butterworth filter and cut into epochs for each participant individually, starting 1,000 ms prior to trial onset until 4,500 ms after trial onset. The envelope was then calculated on the group average based on the Hilbert transformation with a moving average window of 200 ms. Baseline correction was applied, i.e., the average EEG amplitude in the interval of 1,000 ms prior to trial onset was subtracted. The resulted smoothed envelope is presented in Figure 1 for the electrodes C3 and C4. Here, we clearly see desynchronization effects in C3 for right hand motor imagery and C4 for left hand motor imagery starting around 500 ms after trial onset.

We further calculated signed biserial correlation coefficients (*r*^2^) on the smoothed group-average envelope to determine which EEG channels show the most discriminative information for left and right hand motor imagery. Results can be examined in Figure 1 where the scalp patterns of both left and right motor cortex carry relevant class information especially in the beginning of the trial which matches findings in the literature (Pfurtscheller, 1992). Above the scalp patterns, we show the time course over an average of all epochs of the *r*^2^-values for C3 and C4. Here, we can see that on average 500–2,000 ms after trial onset the two channels carry import information to separate right and left motor imagery as indicated by *r*^2^.

We also conducted an offline classification with Common Spatial Patterns (CSP, Ramoser et al., 2000) in comparison to the online classification with Laplacian filters. Individual frequency bands between 8 and 30 Hz and time intervals between 250 and 4,500 ms after stimulus onset were selected for each participant as described in Blankertz et al. (2007). Data was then band-pass filtered in the selected frequency band with a 3rd order zero-phase Butterworth filter and cut into epochs. Six CSP filters were extracted, three per class based on the “ratio-of-median” score as described in Blankertz et al. (2007). The logarithm of the variance of the CSP-filtered signal was then used as features and fed into an RLDA classifier. Overall classification averaged across all participants reached an accuracy of 61.81%. Classification results of CSP vs. Laplacian filters are plotted in Figure 1 (61.81 vs. 57.05%) as well as classification of CSP on *clean* condition vs. the five distraction tasks (67.08 vs. 60.76%).

**Figure 2:**
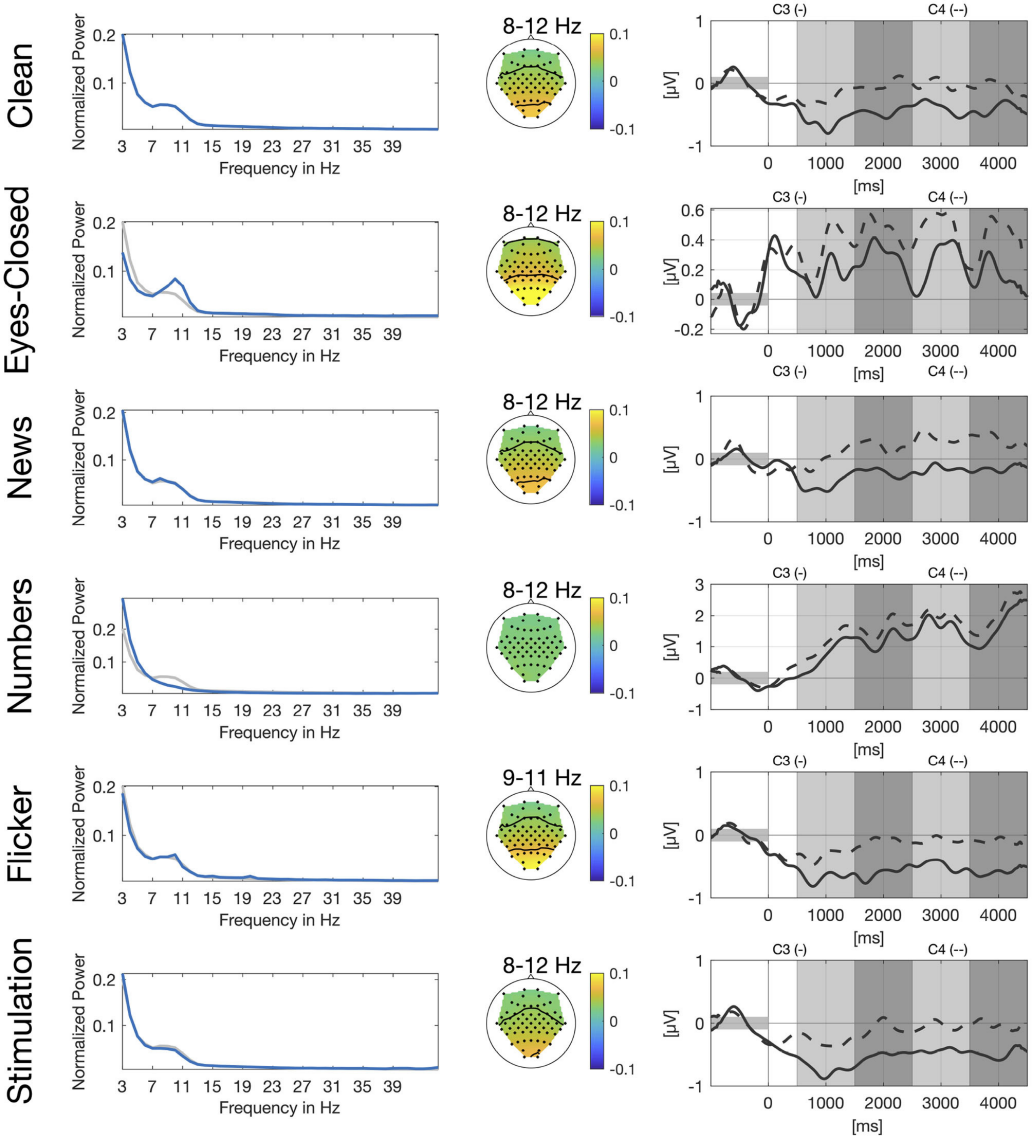
Spectral, spatial, and temporal information for all secondary tasks. Left: Normalized power spectrum averaged across participants and channels O1 and O2, in blue for the respective secondary task and in gray for the clean condition. Center: Spatial distribution of power spectral values averaged across participants for different frequency bands. Right: Group-average envelopes in 9–13 Hz of C3 (solid) and C4 (dashed) electrodes for right hand motor imagery.

In Figure 2, we show power spectra for all secondary tasks. For each participant, power spectra were averaged across trials and normalized channel-wise. We then extracted the power spectra for the channels O1 and O2, averaged over the two channels and again across participants. Alpha peaks clearly differ for *eyes-closed* and *numbers* compared to *clean*. For the *eyes-closed* task, we see the expected alpha peak in the range of 8–12 Hz (Berger, 1929). For the *numbers* task there is no clear alpha peak visible in the occipital channels which is in line with the expected suppression of the visual alpha rhythm during visual search. Power spectrum for the *flicker* task shows a small sharp peak between 9 and 11 Hz which is very close to the frequency of the flickering video and another even smaller peak at 20 Hz which represents the second harmonic of the flicker frequency. The *news* and *stimulation* task do not show clear differences compared to *clean*.

We also show spatial distribution for different frequency bands in the alpha range based on the peaks in the power spectrum. For *eyes-closed* and *flicker* we see a clear activation over the occipital and parietal cortex whereas there is no clear pattern visible for the *numbers* task. Again, patterns for the *news* and the *stimulation* task look very similar to the pattern of the *clean* task.

Similar to Figure 1, we show envelopes of channels C3 and C4 for right hand motor imagery. The modulation of the sensorimotor rhythm is still visible in all conditions as a stronger ERD in C3 compared to C4. However, the effect is obscured by the different artifacts. The disturbences are smallest in the *news, flicker* and the *stimulation* tasks due to the stationary nature of the artifacts. For the *flicker* task we still see a clear difference between both channels, whereas channels are already closer for *eyes-closed* and still even closer for the *numbers* task.

## 3. Conclusion

We recorded a motor imagery-based BCI study with 16 participants where different distraction scenarios are added as secondary tasks to systematically investigate the influence of those noise sources on the motor imagery performance. We have presented group-averages that show typical ERD/ERS effects especially during the first half of the trial over the motor cortex, typical phenomena according to the literature. We further show expected differences in power spectra for occipital channels and spatial patterns for different frequency bands in the alpha range for three of the secondary tasks. We also show classification results of a standard CSP + RLDA classification pipeline that clearly show that classification accuracy decreases in the distraction tasks. All the data^2^ and the code^3^ is publicly available and a more advanced analysis has been published in Brandl et al. (2016).

## Data Availability Statement

The dataset recorded for this study can be found in DepositOnce^1^.

## Ethics Statement

The studies involving human participants were reviewed and approved by Ethikkommission der Charité—Universitätsmedizin Berlin. The patients/participants provided their written informed consent to participate in this study.

## Author Contributions

Design of the study by SB and BB. Recording and analysis of the study by SB. SB wrote the manuscript which was revised by BB. All authors contributed to the article and approved the submitted version.

## Conflict of Interest

The authors declare that the research was conducted in the absence of any commercial or financial relationships that could be construed as a potential conflict of interest.

## Footnotes

^1^https://bnci-horizon-2020.eu/database/data-sets

^2^https://depositonce.tu-berlin.de/handle/11303/10934.2

^3^https://github.com/stephaniebrandl/bci-under-distraction

^4^https://github.com/bbci/bbci_public
